# Measuring Rapid *A–Ci* Curves in Boreal Conifers: Black Spruce and Balsam Fir

**DOI:** 10.3389/fpls.2019.01276

**Published:** 2019-10-25

**Authors:** Carole Coursolle, Guillaume Otis Prud’homme, Manuel Lamothe, Nathalie Isabel

**Affiliations:** ^1^Laurentian Forestry Centre, Canadian Forest Service, Natural Resources Canada, Québec City, QC, Canada; ^2^ Ministère des Forêts, de la Faune et des Parcs, Direction de la recherche forestière, Québec, QC, Canada

**Keywords:** rapid *A–C_i_* curves, boreal conifers, LI-6800, phenotyping, photosynthesis, *V_cmax_*, *J_max_*, phenomics

## Abstract

Climate change is steering tree breeding programs towards the development of families and genotypes that will be adapted and more resilient to changing environments. Making genotype–phenotype–environment connections is central to these predictions and it requires the evaluation of functional traits such as photosynthetic rates that can be linked to environmental variables. However, the ability to rapidly measure photosynthetic parameters has always been limiting. The estimation of *V* *_c,max_* and *J* *_max_* using CO_2_ response curves has traditionally been time consuming, taking anywhere from 30 min to more than an hour, thereby drastically limiting the number of trees that can be assessed per day. Technological advancements have led to the development of a new generation of portable photosynthesis measurement systems offering greater chamber environmental control and automated sampling and, as a result, the proposal of a new, faster, method (RACiR) for measuring *V* *_c,max_*and *J* *_max_*. This method was developed using poplar trees and involves measuring photosynthetic responses to CO_2_ over a range of CO_2_ concentrations changing at a constant rate. The goal of the present study was to adapt the RACiR method for use on conifers whose measurement usually requires much larger leaf chambers. We demonstrate that the RACiR method can be used to estimate *V* *_c,max_* and *J* *_max_* in conifers and provide recommendations to enhance the method. The use our method in conifers will substantially reduce measurement time, thus greatly improving genotype evaluation and selection capabilities based on photosynthetic traits. This study led to the developpement of an R package (RapidACi, https://github.com/ManuelLamothe/RapidACi) that facilitates the correction of multiple RACiR files and the post-measurement correction of leaf areas.

## Introduction

Climate change is steering tree breeding programs towards the development of families and genotypes that will be adapted and more resilient to the warming climate and thereby ensure the health and productivity of forests (Aitken and Bemmels, 2015). Despite the advances in genomics, the prediction of tree responses to future climate remains challenging because of the difficulty in measuring phenotypes related to adaptation. Making genotype–phenotype–environment connections is central to these predictions but the list of traits reflecting tree adaptation to environment that could be efficiently assessed is limited. Indeed, measuring ecophysiological traits can be laborious and time consuming, thus limiting the ability to establish relationships between genotypes and plant response to environmental conditions. Rapid, large-scale screening, or plant phenomics (Furbank and Tester, 2011; Stinziano et al., 2017), are required to overcome these constraints.

Developing indicators for photosynthetic performance are of particular interest when trying to select genotypes that will be adapted to future climates. *V* *_c,max_*, the maximum rate of ribulose-1,5-bisphosphate carboxylation (Rubisco) and *J* *_max_*, the maximum rate of electron transport, are two such parameters (Long and Bernacchi, 2003). These two parameters are also incorporated into Earth System Models (Rogers, 2014) and can be used as “ground truthing” parameters for the development of phenotyping and forest health monitoring platforms using unmanned aerial vehicles (UAVs) and other types of mobile systems in combination with spectral measurements (Dash et al., 2017, Thompson et al., 2018), which is another incentive for the development of a method to obtain reliable estimates rapidly. Estimates of *V* *_c,max_* and *J* *_max_* are obtained through the measurement and modelling of photosynthetic response to CO_2_ concentration (*A–C* *_i_* curves), which until recently could take anywhere from 30 min to more than 60 min per curve (depending on the number of CO_2_ concentrations measured and the system used), thereby greatly limiting the number of measurements that can be made daily.

Recent technological advances have resulted in the development of a new generation of portable photosynthesis measurement systems. With better chamber environmental controls, automation, logging capabilities and computing power, they now permit much greater sampling densities as well as the possibility of accelerating measurement times. Stinziano et al. (2017) have proposed a new method (RACiR) for measuring *A–C* *_i_* curves. By continuously increasing or decreasing the chamber CO_2_ concentration (ramping), they were able to take less than 5 min for each curve. However, this method was developed using poplar leaves (*Populus deltoids* Barr.) and a small chamber (6 cm^2^ leaf aperture), which is not really suitable for measuring most conifers where a much larger chamber (36 cm^2^ leaf aperture in our case) is usually used. The larger leaf chamber volume causes larger differences in response times between the reference and sample chambers due to greater dilution. This, combined with their unique leaf physiology and geometry, requires that the RACiR method be tested on conifers. The objective of the present study was to develop and test the RACiR method on two boreal conifers, balsam fir (*Abies balsamea* (L.) Mill.) and black spruce (*Picea mariana* (Mill.) Britton), growing in a common garden experiment set up to test the effects of heating the seedling growth environment, using a larger chamber.

## Materials and Methods

### Plant Material and Growth Conditions

The study was conducted using 5-year-old black spruce and balsam fir trees planted in a common garden experiment at the Valcartier experimental station in St-Gabriel-de-Valcartier, Québec (N 46°56′59.93″/W 71°29′53.88″). Bare-root seedlings were planted in triangular plots in May and June 2015. Starting in 2016, half the plots were heated from May to October of each year using 1000 W infrared lamps between May and October to produce a growing temperature 2 °C above ambient temperatures. A branch with at least 3 years of growth (2018, 2017, 2016) was sampled from seven different black spruce (BS) and seven different balsam fir (BF) trees, four growing in heated plots and three growing in unheated control plots. Branches were cut, placed in water, recut and inserted into floral water tubes while still underwater to avoid cavitation. Water levels in the floral tubes were monitored throughout the measuring period and the water was topped up as required using a syringe. All measurements were made on site under normal field conditions.

### Gas Exchange Measurements

An LI-6800 portable photosynthesis system (LI-COR Inc., Lincoln, NE, USA) equipped with the Large Leaf and Needle Chamber (36 cm^2^) and the large light source was used for all measurements. The dark respiration (Rd) and CO_2_ response of photosynthesis (*A–C* *_i_*) of 1-year-old needles were measured on site on July 16, 18 and 19, 2018. Two different methods were used to measure *A–C* *_i_*: (1) The traditional method (*A–C* *_i_*-TRAD), involving net assimilation (A_n_) measurements at a predetermined set of CO_2_ concentrations (ex. Long and Bernacchi, 2003) and (2) The rapid method (RACiR), involving continuous measurements over a programmed ramp of changing CO_2_ concentrations (ex. Stinziano et al., 2017). Both a traditional and a RACiR curve were measured on the same set of needles from each shoot. The RACiR curve was measured first followed by the traditional curve. The same environmental conditions were used for both curve types: 1) 22 mmol mol^−1^ of H_2_O in the reference cell producing between ∼63% and 79% relative humidity (RH) in the leaf chamber, depending on the quantity of leaf surface area in the chamber, and producing a variation of ∼2% to 3% in RH over the course of measurements for each shoot; 2) 1200 µmol m^−2^ s^−1^ of light, based on Benomar et al. (2017) and Sendall et al., 2015); 3) chamber air temperature of 25 °C; 4) flow rate of 600 µmol s^−1^; and 5) fan speed of 13,000 rpm.

#### Rapid A–C*_i_* Response Curves

**Figure 1:**
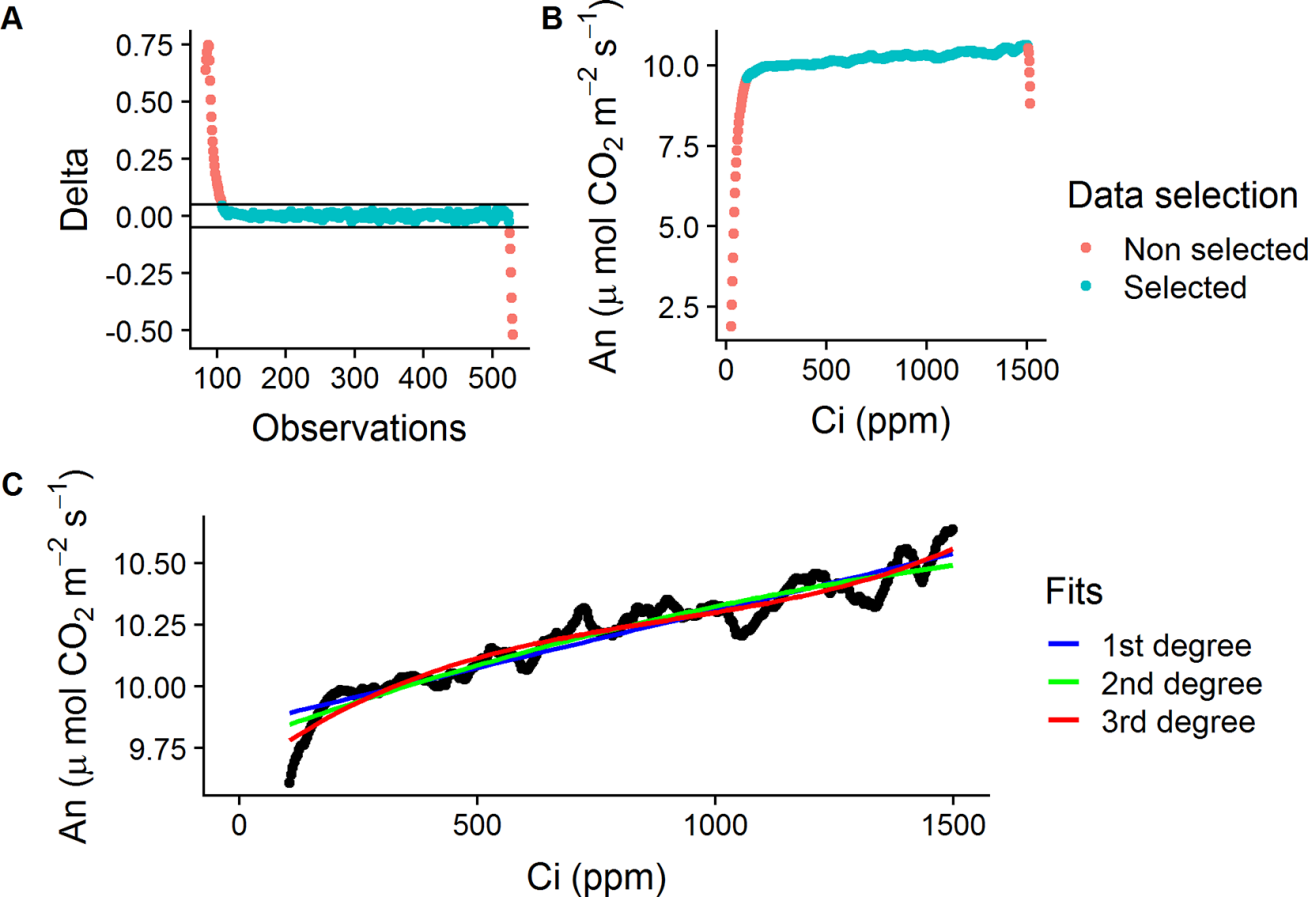
Data selection process for the empty chamber (ECRC) and RACiR response curves. **(A)** Calculated delta values ( ± 0.05, An_i −_ An_i−1_) for a selected ECRC, red symbols indicate outliers, blue symbols indicate selected points. **(B)** A_n_ vs C_i_ for a selected ECRC curve, red symbols indicate non-steady state observations, blue symbols indicate selected points. **(C)** First, second and third degree polynomials fitted to a selected ECRC for correction of raw RACiR data.

The method used for the RACiR curves was adapted from the one developed by Stinziano et al. (2017) for *P. deltoides* Barr. leaves using the LI-6800 Portable Photosynthesis System and the Multiflash Fluorometer and Chamber (LI-COR Inc., Lincoln, NE, USA). Shoots were placed in the Large Leaf and Needle Chamber at 420 ppm CO_2_ concentration ([CO_2_]) and allowed to acclimate for ∼5 min. The LI-6800’s Autocontrol function was used to program a “down” ramp from 420 to 20 ppm at a rate of 200 ppm min^−1^ CO_2_ and was immediately followed, approx. 10 to 15 s later, by an “up” ramp from 20 to 1,520 ppm at a rate of 100 ppm min^−1^. The LI6800’s Autolog feature was used to record measurements every 2 s. The reference and sample infrared gas analyzers (IRGAs) were matched before the start of each curve. Only the portion of data collected from the “up” ramps (20 to 1,520 ppm) were used to establish the CO_2_ response curves. The raw data from these “up ramps” was filtered automatically using a delta threshold value (± 0.05, An_i −_ An_i−1_) to keep only the quasi-linear portion of the data, where the chamber mixing is at steady-state, and to also remove outliers (ex: **Figures 1A, B**).

The raw data obtained from the RACiR curve measurements must be corrected to account for measurement lags between the reference and sample [CO_2_] (**Supplementary Figure 1**) caused by the mixing volume of the chamber, match offsets and system residual time delays (Stinziano et al., 2017). Data collected from the quasi-linear portion of a RACiR curve measured with the chamber empty (ECRC) was used for this correction. Following Stinziano et al. (2017) and Stinziano et al. (2018), we fitted 1st, 2nd and 3rd degree polynomials (**Figure 1C**) to this data and the best fitting model according to the Bayesian information criterion (BIC) was used to correct the quasi-linear portion of the RACiR curve measurements. The script used to make the corrections is available on Github (https://github.com/ManuelLamothe/RapidACi) it can be used to automatically correct multiple files at a time and to carry out post-measurement corrections to leaf area, which are required when measuring non-flat leaves. An example of the analysis can be found in the **Supplementary Material** (**Supplementary Data Sheet 1)**. ECRCs were carried out at the beginning, middle and end of each day using the same environmental conditions as for the actual measurements. Three sets of corrected response curve data, one for each empty chamber, were generated for each measured shoot. Each set of response curve data was corrected using empty chamber data obtained the same day.

#### Traditional A–C*_i_* Response Curves

The LI-6800 CO_2_ response program was used for the *A–C* *_i_*–TRAD curves and the reference CO_2_ concentrations were set to 420, 40, 20, 40, 60, 80, 100, 150, 200, 300, 420, 500, 700, 900, 1,100, 1,300, and 1,500 ppm. Minimum and maximum wait times were set at 60 to 120 s based on the stability of the CO_2_ net assimilation rate and the difference between sample and reference CO_2_ concentrations. The reference and sample infrared gas analyzers (IRGAs) were matched before the measurement at each concentration. The measured shoot was allowed to acclimate at 420 ppm for ∼5 min. The *A–C* *_i_*–TRAD curve was measured on the same needles immediately following the end of the RACiR curve.

#### Dark Respiration (R***_d_Meas_***) Measurements

Shoots were removed from the chamber immediately following the *A–C* *_i_*–TRAD measurement and dark adapted for at least 30 min by covering the measured shoot with aluminum foil. Shoots were then placed in the darkened LI6800 chamber and allowed to acclimate at 420 ppm [CO_2_], 25 °C and 22 mmol mol^−1^ of H_2_O in the reference before recording *R* *_d_meas_*.

### Parameter Estimation and Statistical Analysis

#### Timing of Empty Chamber Response Curve (ECRC)

Corrected CO_2_ response curve data were fitted with the FvCB model using the fitaci function from the Plantecophys R Package (Duursma, 2015) with the default settings and *R* *_d_meas_*as an input. *V* *_c,max_*_,_ *J* *_max_*, and root mean square error (RMSE) were obtained for the three corrected curves (one for each ECRC) from each measured shoot. An analysis of variance on the effect of the timing of the empty chamber measurement series used to correct the RACiR curves was conducted using the nlme R Package (Pinheiro et al., 2018) for mixed models. The analysis was carried out on daily means of *V* *_c,max_*_,_ *J* *_max_*, and RMSE, measurement day was considered a random effect and empty chamber timing (morning, mid-day and afternoon) was considered a fixed effect.

#### Comparison of Traditional and Rapid CO_2_ Response Curve Parameters

*V* *_c,max_* and *J* *_max_* were generated for the *A–C* *_i_*–TRAD and RACiR curves by fitting the FvCB model using the Plantecophys R package (Duursma, 2015) with the default settings of the fitaci function and *R* *_d_meas_* as an input. A third set of parameters was generated using the portion of the measured RACiR curve between 200 and 800 ppm of intercellular CO_2_ concentration (*C* *_i_*) (RACiR–Partial). An analysis of variance for repeated measures on the effect of the curve type was conducted using the nlme R Package (Pinheiro et al., 2018). Curve type was the repeated factor, tree was considered as a random effect and species, curve type and their interaction were considered fixed effects. The heating effect (main and interactions with curve type) was included as a fixed effect in the original ANOVA model but found to be non-significant (*V* *_c,max_*: F = 0.6604, p = 0.4334 for the main effect and F = 2.4311, p = 0.1135 for the interaction; *J* *_max_*: F = 1.2578, p = 0.2883 for the main effect and F = 1.7539, p = .1987 for the interaction) so it was removed from the final model.

Dark respiration estimates were also obtained using the Plantecophys R package (Duursma, 2015), with its default settings for each curve type, so as to provide additional information on the performance of the three curve fitting methods. An analysis of variance on the effect of the method used to estimate dark respiration, measured (*R* *_d_meas_*) or estimated using *A–C* *_i_*–TRAD (*R* *_d_aci_*), RACiR (*R* *_d_racir_*) and RACiR–Partial (*R* *_d_part_*), was conducted using the same parameters as for *V* *_c,max_* and *J* *_max_*. Again, the heating effect (main and interactions with measurement type) was included as a fixed effect in the original ANOVA model but found to be non-significant (F = 1.1313, p = 0.3125 for the main effect and F = 0.0782, p = 0.9713 for the interaction) so it was removed from the final model.

## Results

### Timing of Empty Chamber Response Curve Measurements (ECRC)

**Figure 2:**
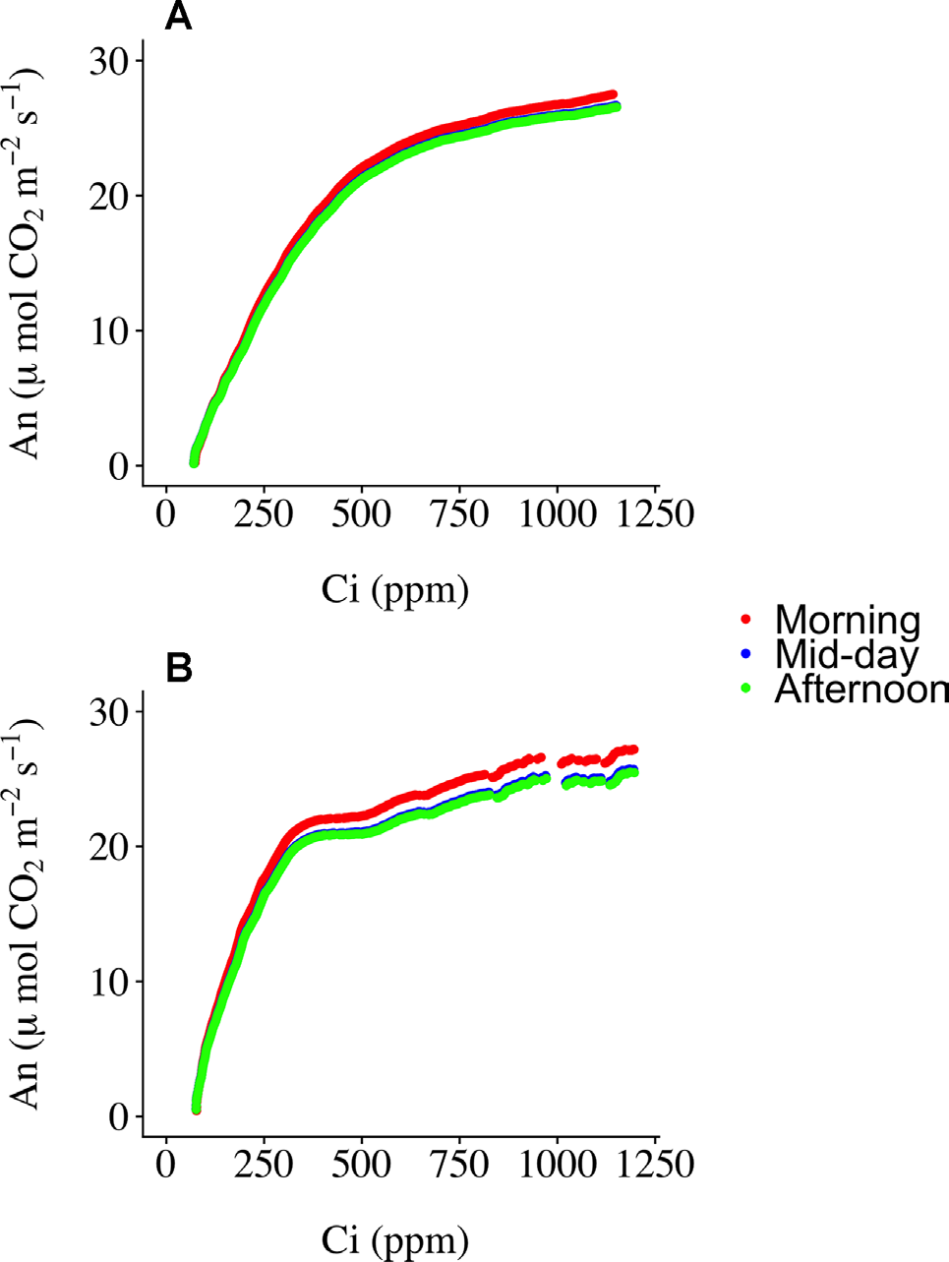
Corrected RACiR curves using the morning, mid-day and afternoon empty chamber response curves (ECRC) for a selected balsam fir **(A)** and black spruce **(B)** shoot.

Three ECRCs were generated per day in an attempt to determine how often an empty chamber response curve needed to be generated to correct the raw RACiR curve measurements. The analyses of variance indicated that the timing of the generation of the ECRC had no significant effect on *V* *_c,max_*(F = 0.7588, p = 0.5256) generated using the F_v_CB model. However, ECRC timing had a significant effect on *J* *_max_* (F = 7.8493, p = 0.0412), values generated using the morning ECRC being significantly higher (p < 0.05) than those generated using the mid-day ECRC (**Table 1**). Finally, RMSE of the fitted F_v_CB models were not significantly affected by the timing of the ECRC (F = 1.25961, p = 0.3765, **Table 1**). **Figure 2** illustrates the correction effect on the net assimilation rates for a selected balsam fir and black spruce tree. The differences appear to be minimal. The mid-day empty chamber response curve was chosen to correct the RACiR curves for the comparison between traditional and rapid response curves since it was the measurement that was the closest in timing to the largest number of RACiR curves. Furthermore, the *J* *_max_* generated by the morning empty chamber was the only parameter that was significantly different from the mid-day and afternoon empty chamber curves.

**Table 1:** LSmeans of *V* *_c,max_*, *J* *_max_* and RMSE generated using morning, mid-day and late afternoon empty chamber corrections are shown.

ECRC	*V* *_c,max_* ******(µmol m^−2^ s^−1^)		*J* *_max_* ******(µmol m^−2^ s^−1^)		RMSE	
	Mean	SE	Mean	SE	Mean	SE
Morning	71.1841^a^	4.5118	127.2851^a^	5.9813	10.4003^a^	1.4394
Mid-day	64.1333^a^	4.5118	119.8836^b^	5.9813	9.8551^a^	1.4394
Late Afternoon	70.6677^a^	4.5118	121.2287^a,b^	5.9813	10.7410^a^	1.4394

Means followed by the same letter are not significantly different (p < 0.05) for the variable in question.

### Comparison of Traditional and Rapid CO_2_ Response Curves and Parameters

**Figure 3:**
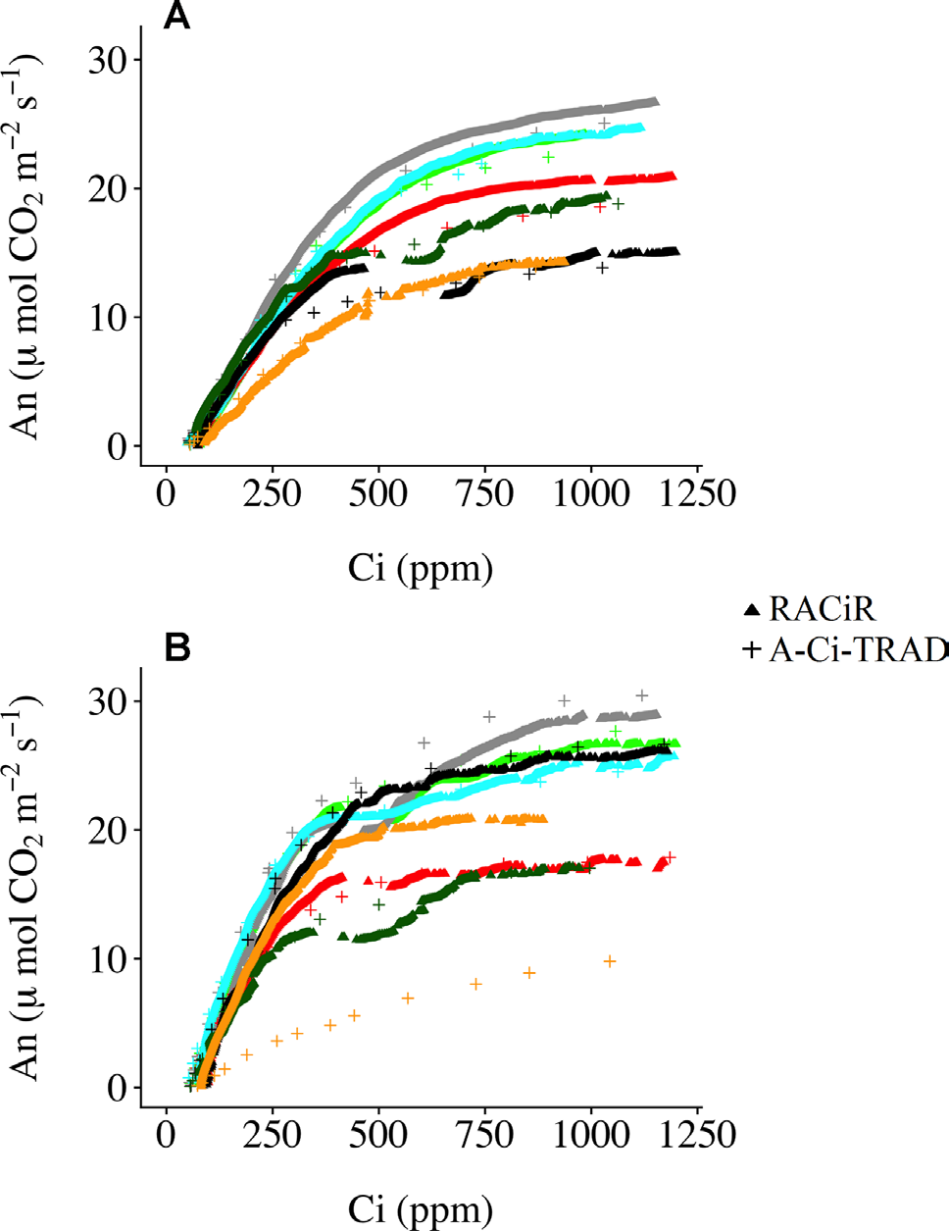
Traditional (*A–C* *_i_*–TRAD) and rapid (RACiR) CO_2_ response curves for the seven balsam fir **(A)** and seven black spruce **(B)** shoots measured.

**Figure 4:**
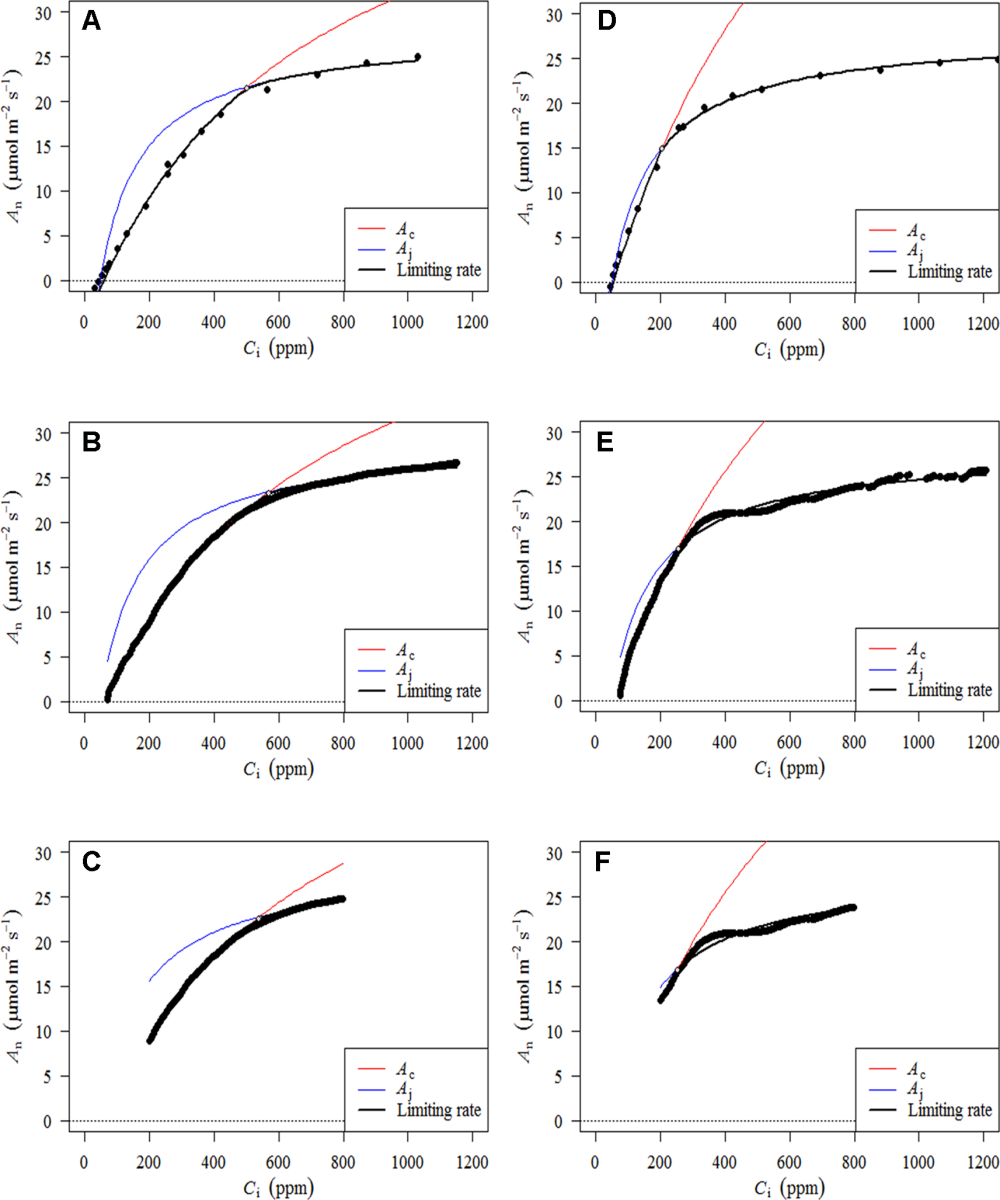
Fitted FvCB models using *A–C* *_i_*–TRAD **(A**, **D)**, RACiR **(B**, **E)** and RACiR–Partial **(C**, **F)** data for a selected Balsam fir **(A**, **B**, **C)** and black spruce **(D**, **E**, **F)** shoot. The graphs were generated by the fitaci function of the Plantecophys R package (Duursma, 2015) using the default settings and *R* *_d_meas_* as an input.

**Figure 3** presents *A–C* *_i_*–TRAD and corrected RACiR curves for the balsam fir (a) and black spruce (b) shoots measured. Generally, there is good agreement between the two types of curve when *C* *_i_* is below 500 ppm and where *d*An/*dC* *_i_* is high. However, there is greater variability and deviation between the two types of curves at higher *C* *_i_* concentrations. Also, small drops in net assimilation within the approximate range of 450–600 ppm *C* *_i_* can be seen for the RACiR curves of some trees. These data points were considered erroneous and removed before fitting the FvCB model. **Figure 4** presents the fitted FvCB model using *A–C* *_i_*–TRAD (a, d), RACiR (b, e) and RACiR–Partial (c, f) data for a selected balsam fir (a, b, c) and black spruce (d, e, f) shoot. RMSEs of the fitted FvCB models ranged from 2.224 to 13.746 for the *A–C* *_i_*–TRAD curves, from 3.834 to 21.787 for the RACiR curves and from 3.243 to 15.065 for the RACiR–Partial curves.

An ANOVA conducted for the response curve parameters (*V* *_c,max_*, *J* *_max_*) and RMSE of the fitted models indicated that the response curve type did not significantly affect either *V* *_c,max_*or *J* *_max_*,**but the RMSE of the models were significantly different (**Tables 2**, **3**). The RMSEs of the fitted RACiR and RACiR–Partial curves were significantly but only slightly to moderately higher than the RMSE of the fitted *A–C* *_i_*–TRAD curve.

**Table 2:** Results of the ANOVA for the comparison of *V* *_c,max_*, *J* *_max_* and RMSE generated by the FvCB model using the three different response curve methods.

Fixed Effect	Num. DF	Den. DF	V_c,max_		J_max_		RMSE	
			F	p	F	p	F	p
Species	1	23	6.640	0.0242	1.763	0.2090	4.162	0.0640
Curve Type (CT)	2	23	0.006	0.9940	1.604	0.2220	28.084	<0.0001
Species*CT	2	23	0.051	0.9501	0.004	0.9961	0.081	0.9227

Num. DF, numerator degrees of freedom; Den. DF, denominator degrees of freedom.

**Table 3:** LSmeans for *V* *_c,max_*, *J* *_max_* and RMSE generated by the 3 different types of curves.

Curve type	*V* *_c,max_* ******(µmol m^−2^ s^−1^)		*J* *_max_* ******(µmol m^−2^ s^−1^)		RMSE	
	Mean	SE	Mean	SE	Mean	SE
*A–C* *_i_*–TRAD	62.09^a^	1.09	112.60^a^	8.34	3.73^a^	1.14
RACiR	62.02^a^	1.09	119.59^a^	8.34	8.59^b^	1.14
RACiR–Partial	61.59^a^	1.09	118.42^a^	8.34	6.10^c^	1.14

Means followed by the same letter are not significantly different (p < 0.05) for the variable in question.

The statistical analyses indicate that dark respiration was significantly affected (F = 5.4684, p = 0.0033) by the estimation method used. The *A–C* *_i_*–TRAD curve yielded the lowest dark respiration (*R* *_d_aci_*) value while *R* *_d_meas_* was the highest. *R* *_d_aci_* was the only dark respiration measurement that was significantly different from *R* *_d_meas_* (**Table 4**). *R* *_d_part_* was quite similar to *R* *_d_aci_*while *R* *_d_racir_* was closer to *R* *_d_meas_*.

**Table 4:** LSmeans for measured dark respiration (*R* *_d_meas_*) and dark respiration estimated from the *A–* *C* *_i_*–TRAD (*R* *_d_aci_*), RACiR (*R* *_d_racir_*) and RACiR–Partial (*R* *_d_part_*) response curves.

	Dark Respiration	
Measurement Type	Mean (µmol m^−2^ s^−1^)	SE
Measured	2.19^a^	0.40
*A–C* *_i_*–TRAD	0.52^b^	0.40
RACiR	1.85^a,b^	0.40
RACiR–Partial	0.78^a,b^	0.40

Means followed by the same letter are not significantly different (p < 0.05).

## Discussion

### LI-6800 Configuration Considerations for Conifer Needle Measurements

Compared to previously published studies describing the RACiR method applied to flat leaves (Stinziano et al., 2017; Pilon et al., 2018), the needle-leaf geometry of conifer species requires a different instrument configuration for photosynthesis measurements. A much larger leaf chamber (193.7 cm^3^, large needle and leaf chamber, vs 87.3 cm^3^ for the fluorometer chamber, in our case) is generally used, thereby leading to a greater “lag time” required for the differences between the sample and reference [CO_2_] to stabilize compared to the smaller chambers. We noted “lag times” of between 48 and 92 s. (Mean = 63.4, SE = 4.0) compared to the 20–30 s reported by Stinziano et al., 2017 for poplar leaves. The larger lag times caused by the larger cuvette mean that a greater number of potential data points at the start of the ramp (low *Ci* concentrations in our case) are lost, so preliminary tests should always be carried out to adequately identify the starting CO_2_ concentration for the ramp. The same should apply for the identification of the ramp end point.

Preliminary tests using a controlled target leaf VPD revealed the existence of periodic noise in the empty chamber measurements caused by adjustments in the H_2_O control loop and the large chamber volume (Pers. Comm: D. Lynch, LI-COR Inc., Lincoln, NE, USA). This led at times to unrealistic, negative *C* *_i_* values and was remedied by controlling the H_2_O content of the air in the reference cell instead. Preliminary tests allowed us to choose a target concentration (22 mmol mol^−1^) which produced RHs between ∼63 and 79% in the leaf chamber, thereby assuring that shoot samples were not stressed. This also produced stomatal conductances that were high enough to minimize noise in the H_2_O analyzer, which minimized noise in the calculated *C* *_i_* values (Pers. Comm: D. Lynch, LI-COR Inc., Lincoln, NE, USA).

### Timing of Empty Chamber Response Curve Measurements (ECRC)

Determining the best moment to conduct the ECRC so as to keep the number of required ECRCs to a minimum is an important factor given that one of the main reasons for using the RACiR method is reducing the overall measurement time for producing photosynthetic parameters that can be used for phenotyping and health/stress monitoring. Three ECRCs were measured daily, one in the morning before the first shoot measurement, one around mid-day and one in the late afternoon after the last shoot measurement of the day. The *J* *_max_* generated by the morning ECRC was the only parameter that was significantly different among the ECRCs (**Table 1**) and the A_n_ vs *C* *_i_* relationships obtained from the three different corrected RACiR curves were quite similar (**Figure 2**). The significantly higher *J* *_max_* generated by the morning ECRC may have resulted from the fact that we matched the reference and sample IRGAs before measuring each RACiR curve. In a letter published after the conclusion of our study, Stinziano et al., 2018 recommend using the same match for the ECRC and RACiR curves. In fact, we noticed that the largest match adjustments tended to occur towards the beginning of the day and would stabilize thereafter, which most likely explains the lack of significant differences between the RACiR parameters calculated using the mid-day and late afternoon ECRCs. Given our results, we feel that it is most likely feasible to conduct only one ECRC per measurement run per day as long as matches are checked regularly, remain constant and chamber settings do not change. It would also probably be best to run the LI-6800 at the desired chamber settings for several minutes and conduct more than one match before starting a measurement run.

### RACiR Vs Traditional CO_2_ Response Curves

**Figure 3** presents the corrected “raw” response curve data generated by the RACiR and *A–C* *_i_*–TRAD methods for the seven balsam fir a) and seven black spruce b) shoots. With the exception of one black spruce shoot, there is generally good agreement when *d*An/*dC* *_i_* is high (at *C* *_i_* values below ∼500 ppm), the area related to Rubisco activity (*V* *_c,max_*), but there is more variability at higher *C* *_i_*values related to RuBP regeneration (*J* *_max_*; Long and Bernacchi, 2003). These results are generally similar to those reported by Stinziano et al. (2017) and Taylor and Long (2018), using an LI-6800, and to Bunce, 2018, using a CIRAS-3. We believe that the divergence between the two response curve types for one of the black spruce shoots may be linked to a problem with the *A–C* *_i_*–TRAD measurement and not the RACiR method, given the unusual form of the *A–C* *_i_*–TRAD curve compared to the others. Contrary to Taylor and Long (2018), we did not observe significant offsets for CO_2_ compensation points. CO_2_ compensation points calculated using RACiR data were generally higher (62.5 without *R* *_d_meas_*and 70.6 using *R* *_d_meas)_* compared to *A–C* *_i_*–TRAD (51.1 without *R* *_d_meas_*and 68.1 using *R* *_d_meas_*) data but not significantly different (p = 0.06 without *R* *_d_meas_* and 0.39 using *R* *_d_meas_*). This would seem to confirm the assertion by Stinziano et al. (2018) that ramp rates above 100 ppm min^−1^ can be problematic. Using measured values for dark respiration seems to reduce offsets.

Our results (**Table 4**) indicate that the dark respiration (*R* *_d_*) estimated by the FvCB model using the RACiR method (1.85 µmol m^−2^ s^−1^) was the closest estimate to, and not significantly different from, *R* *_d_meas_* (2.19 µmol m^−2^ s^−1^). Furthermore, although considerably lower, *R* *_d_*estimated using the RACiR–Partial method (0.78 µmol m^−2^ s^−1^) was not significantly different from *R* *_d_meas_*. The *R* *_d_*estimated using the *A–C* *_i_*–TRAD method (0.52 µmol m^−2^ s^−1^) was considerably lower and significantly different from *R* *_d_meas_*. These results indicate that the RACiR method performs very well when used to estimate dark respiration and this better performance may be linked to the much larger number of data points available for the estimate compared to *A–C* *_i_*–TRAD data.

We found that there were no significant differences among the *V* *_c,max_*and *J* *_max_* values generated by the traditional or either of the two rapid response curve methods (**Tables 2** and **3**) when *R* *_d_meas_* was used as an input to the FvCB model. *V* *_c,max_* values ranged from 61.59 to 62.02 µmol m^−2^ s^−1^ (SE = 1.09) and *J* *_max_* values ranged between 112.6 and 119.59 µmol m^−2^ s^−1^ (SE = 8.34). However, a separate ANOVA looking at *V* *_c,max_*and *J* *_max_* generated without using *R* *_d_meas_* as an input revealed that the curve fitting method had a significant effect on the *J* *_max_* generated (F = 5.3946, p = 0.0116), the RACiR method (Mean = 117.63, SE = 8.7215) producing a significantly greater *J* *_max_* compared to the *A–C* *_i_*–TRAD method (Mean = 103.02, SE = 8.72). This result is not surprising given that the *A–C* *_i_*–TRAD method generated dark respiration estimates that were significantly lower than the measured values. Furthermore, an ANOVA conducted to analyze the effect of using *R* *_d_meas_* vs estimated *R* *_d_* on the generation of *V* *_c,max_*and *J* *_max_* values by the *A–C* *_i_*–TRAD method revealed that using *R* *_d_meas_*had a significant (*V* *_c,max_*: F = 11.1819, p = 0.0074; *J* *_max_*: F = 54.7402, p < 0.0001) effect.

These results clearly show that the RACiR method can be used for a larger chamber size required by conifers and that measuring dark respiration so as to provide the most accurate input to the FvCB model improves the estimated *J* *_max_*. Also, we believe that using the RACiR method when *R* *_d_meas_* is not available would produce the best estimates of *V* *_c,max_*and *J* *_max_* given that this method provides an estimate of dark respiration that is much closer to, and not significantly different from, the measured dark respiration when compared to the *A–C* *_i_*–TRAD method.

Results concerning statistical comparisons involving the values generated by the RACiR–Partial method should be interpreted with caution. We did not generate a complete independent set of CO_2_ response curves for this method but, instead, used a subset of the data that were generated by the RACiR curves. This may have led to some autocorrelation among the data, but we believe that the potential of using smaller [CO_2_] ranges for CO_2_ response curves of conifers should be further investigated, the smaller the required range, the faster the measurement.

## Conclusions and Recommendations

1. The RACiR method can be used for measuring conifers using large needle and leaf chambers, 193.7 cm^3^ in our case. The exact length of the ramp as well as the appropriate chamber environmental conditions need to be identified during pre-tests and may vary depending on the species and size of chamber used.
2. Typically, a traditional *A–C* *_i_* takes between 30 and 60 min, approx. 30 to 36 min (excluding acclimation time) in our case, depending on the number of points and wait times at each CO_2_ concentration. A total maximum time of approximately 22 min was required to measure a full RACiR curve using the method we have developed. However, as shown by our analysis of the partial RACiR curves (between 200 and 800 ppm internal CO_2_ concentration), we believe that total measurement time could be reduced by at least 50% by reducing the ramp range. This method could be used to obtain “ground truthed” photosynthetic capacity estimates to validate air-borne spectral measurements and thereby accelerate and facilitate the development of plant stress and phenotyping platforms.
3. As suggested by Stinziano et al. (2018), we believe that carrying out an empty chamber response curve measurement (ECRC) to correct the RACiR measurements should be carried out for each RACiR measurement series, i.e. at least once a day, and not necessarily after each RACiR measurement, and when environmental conditions in the chamber change. Our findings indicate that the timing is less important as long as the match adjustment remains constant for all of the measured curves in the series.
4. Whenever possible, we recommend measuring dark respiration independently so as to produce the best possible estimates of *V* *_c,max_*and *J* *_max_*. This could be achieved by using a second photosynthesis measuring system so as to decrease measurement time.

## Data Availability Statement

The datasets generated for this study are available on request to the corresponding author. Also, data from one uncorrected RACiR curve and its corresponding ECRC and ACi-TRAD are provided at https://github.com/GuillaumeOtisPrudhomme/TestFiles for use with the example analyses in Data Sheet 1.

## Author Contributions

CC, with input from GP, contrived the study and wrote the manuscript. GP produced the figures. GP and CC collected and analyzed the data. ML provided input to the statistical analysis. ML and GP developed the scripts used to correct the RACiR curves with input from CC. GP and NI read and commented on the manuscript.

## Funding

Funding was provided to NI by the Genomics Research and Development Initiative (GRDI) of the government of Canada.

## Conflict of Interest

The authors declare that the research was conducted in the absence of any commercial or financial relationships that could be construed as a potential conflict of interest.
