# Supplementary material for: Measuring Rapid A–Ci Curves in Boreal Conifers: Black Spruce and Balsam Fir
Source: Front Plant Sci. 2019 Oct 25;10:1276. doi: 10.3389/fpls.2019.01276 (PMC6823239; doi:10.3389/fpls.2019.01276)

Supplementary Material

Article title: Measuring rapid A-C_i curves in boreal conifers: Black spruce and balsam fir

Authors: Carole Coursolle, Guillaume Otis Prud'homme, Manuel Lamothe, Nathalie Isabel

Supplementary Figure 1: Figure demonstrating the lag in CO_2 concentrations between the reference (red) and sample (blue) cells over the measurement period for a selected shoot.

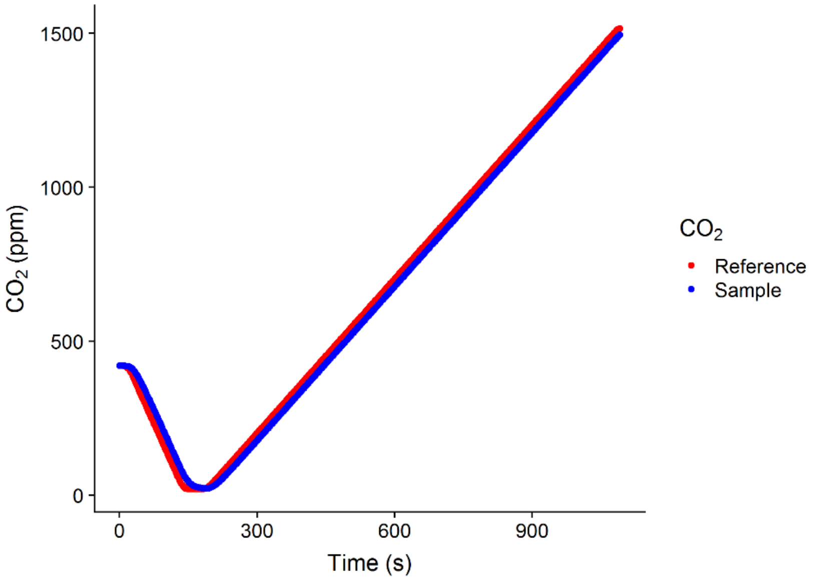

Supplement: Supplementary file 1 [file Image_1.pdf]
