# Supplementary material for: Measuring Rapid A–Ci Curves in Boreal Conifers: Black Spruce and Balsam Fir
Source: Front Plant Sci. 2019 Oct 25;10:1276. doi: 10.3389/fpls.2019.01276 (PMC6823239; doi:10.3389/fpls.2019.01276)
Supplement: Supplementary file 2 [file DataSheet_1.pdf]

Rapid A-Ci example

Installation and setup

```
devtools::install_github("ManuelLamothe/RapidACi")
```

```
if(!require(tidyverse))install.packages("tidyverse")
if(!require(readxl))install.packages("readxl")
if(!require(httr))install.packages("httr")
if(!require(XLConnect))install.packages("XLConnect")
if(!require(plantecophys))install.packages("plantecophys")


library(RapidACi); library(readxl); library(httr); library(tidyverse); library(XLConnect); library(plantecophys);
```

Using the package RapidACI

```
dir.create("data/", showWarnings = FALSE)
testfile.url <- "https://github.com/GuillaumeOtisPrudhomme/TestFiles/raw/master/"
httr::GET(url = paste0(testfile.url, "Fast.xlsx"), write_disk("data/Fast.xlsx", overwrite = TRUE))
```

```
httr::GET(url = paste0(testfile.url, "Empty_2.xlsx"), write_disk("data/Empty.xlsx", overwrite = TRUE))
```

```
list_files <- build_list()

list_files$sample_ID[1] <- "fastdemo"

results <- Rapid_aci_correction(list_files)

# the following command produces plot 1, 2, and 3; plot 4 is produced with the argument priority_curve  set to
"negative". The plots are saved to a file in the figure/ directory.

diagnose_sample(results, sample_name = "fastdemo")
```

Plot 1 : Viewing the selected delta : data/Empty.xlsx

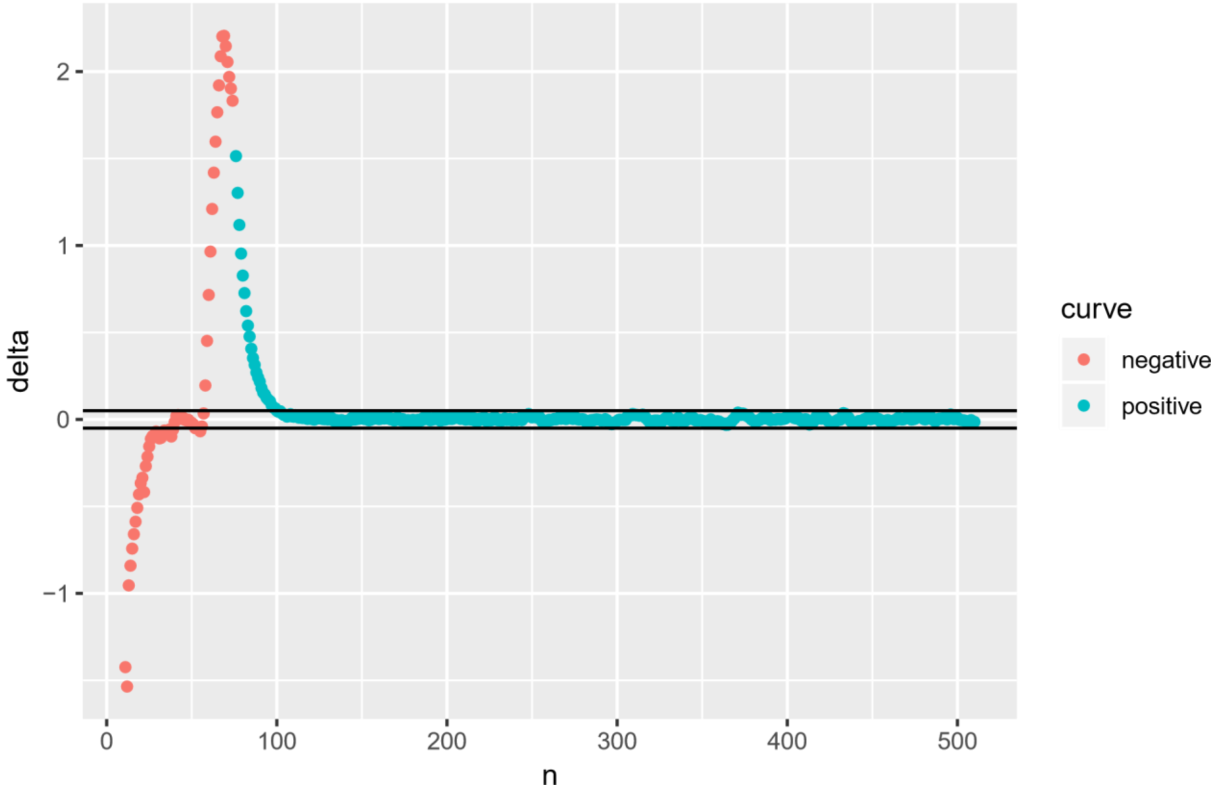

Plot 2 : Portion of curve analyzed : data/Empty.xlsx

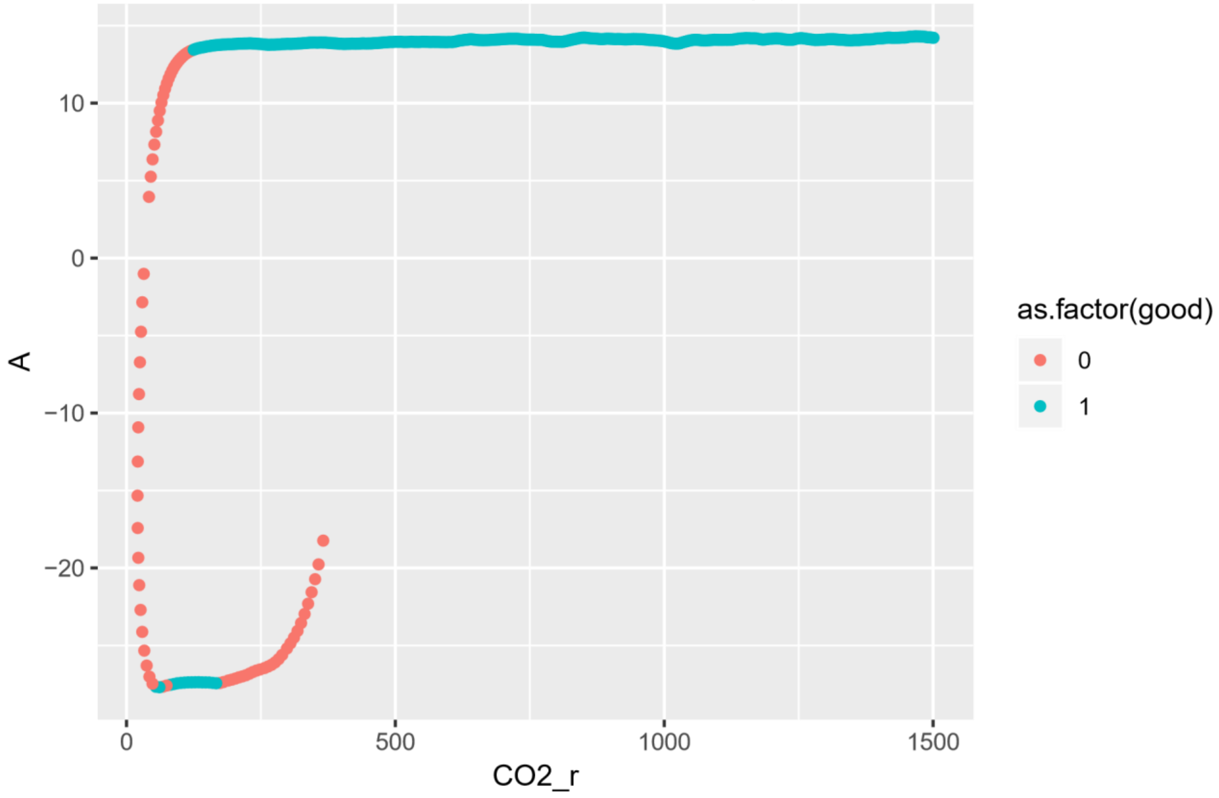

Plot 3 : Positive curve : data/Empty.xlsx

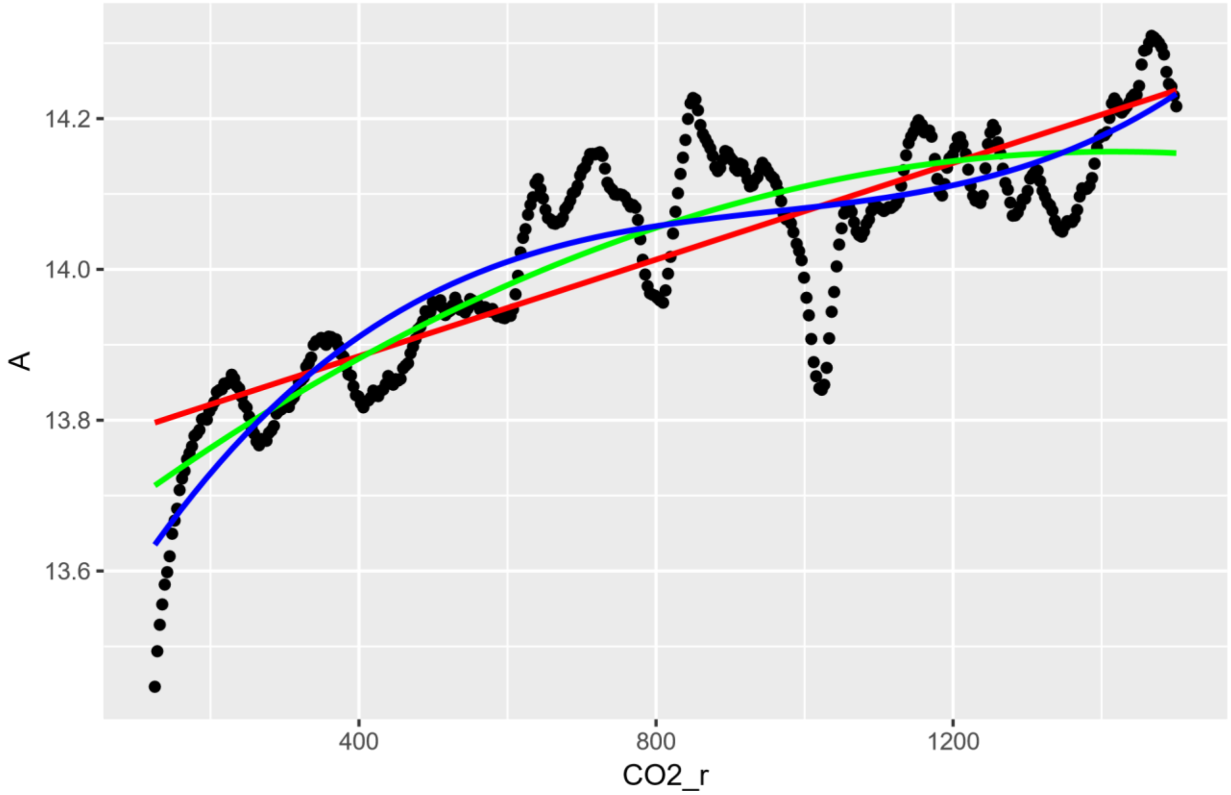

Plot 4 : Negative Curve : data/Empty.xlsx

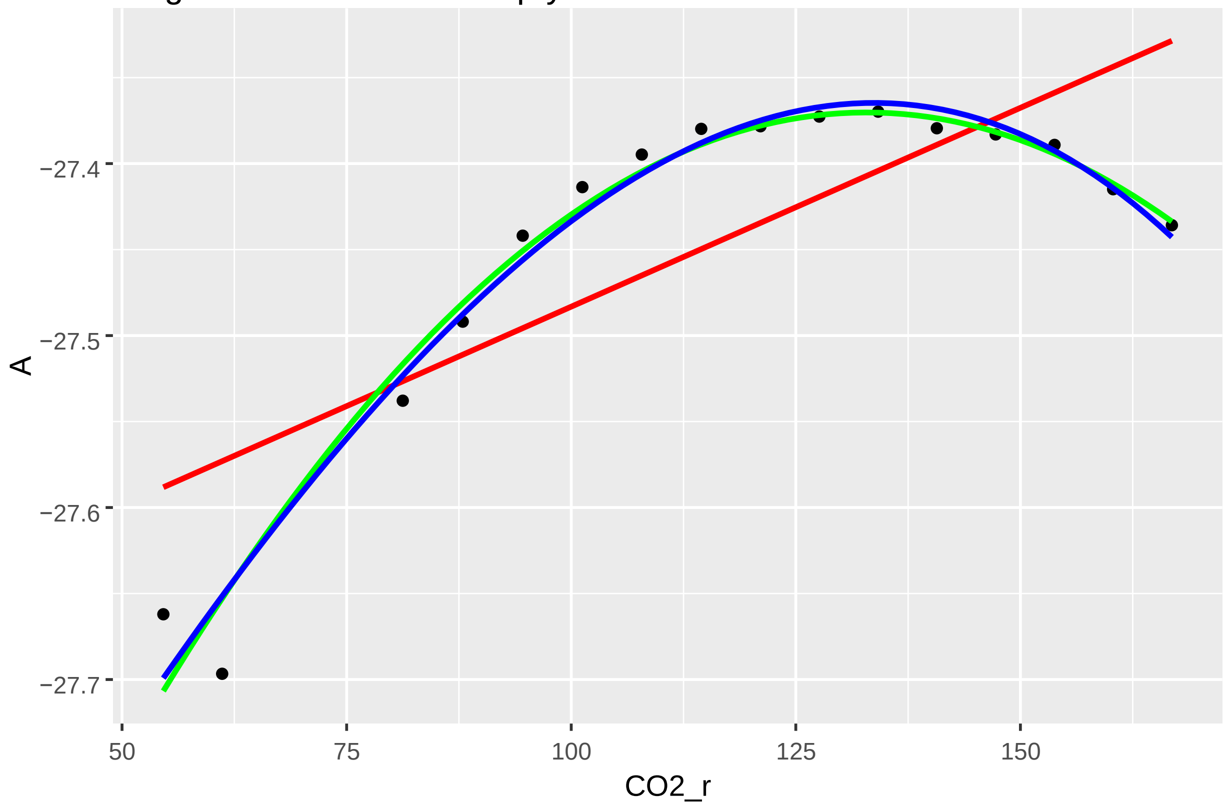

ANALYSIS OF THE RESULTING CORRECTED RAPID A-CI CURVE

Corrected Rapid A-Ci

```
Raci <- results[["fastdemo"]]$Raci
```

Import Dark respiration from Dark file

```
httr::GET(url = paste0(testfile.url, "Dark.xlsx"), write_disk("Dark.xlsx", overwrite = TRUE))
Dark<- read_excel("Dark.xlsx", range="Measurements!I17", col_types = "numeric", col_names = FALSE)

Raci$Rd <- pull(Dark)
```

Fit rapid A-Ci with plantecophys

```
fRaci <- fitaci(Raci, useRd=TRUE)
par(mgp=c(2.5,1,0), family="serif", ps=14)
plot(fRaci, las=1,xlim=c(0,1200), ylim=c(0,30))
```

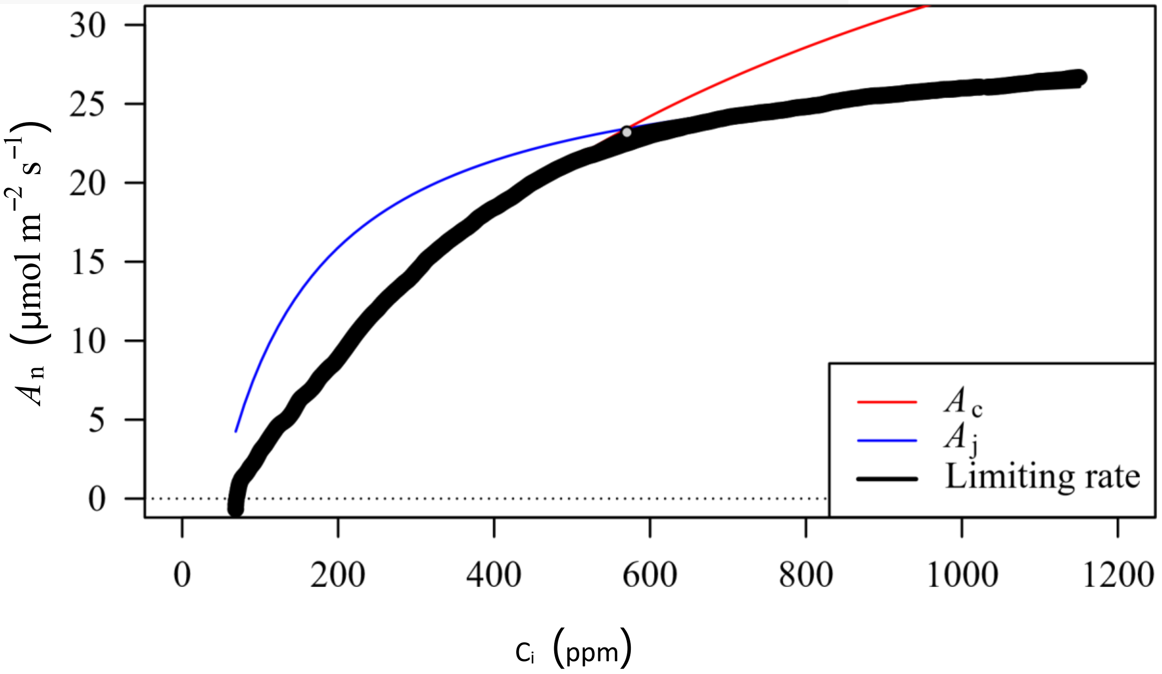

```r
# Estimation of Co2 Compensation point
CP_Raci <- fRaci$Ci(0)
```

Extracting partial curve (between 200 to 800 ppm)

```r
RaciPartial <- subset(Raci, Raci$Ci>=200 & Raci$Ci<=800, c(Photo, Ci, PARi, Tleaf, GasEx_gsw, Rd))
```

```r
# Refit partial curve
fRaciPartial <- fitaci(RaciPartial, useRd =TRUE)
par(mgp=c(2.5,1,0), family="serif", ps=14)
plot(fRaciPartial, las=1,xlim=c(0,1200), ylim=c(0,30))
```

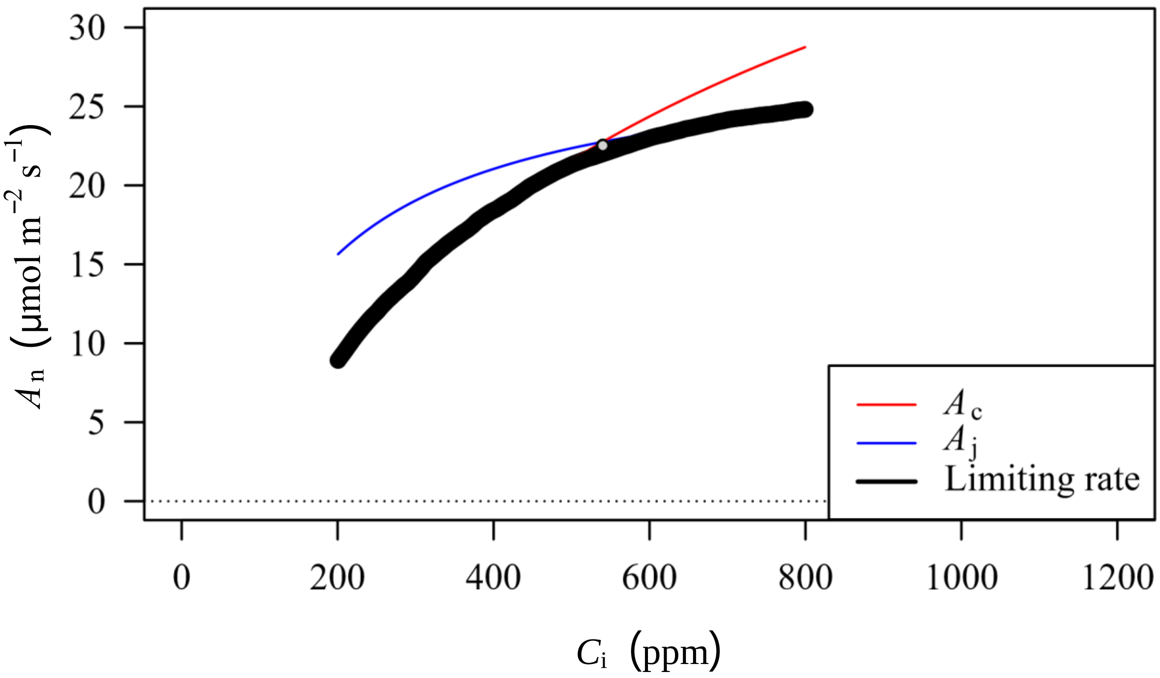


```r
# Estimation of Co2 compensation point from partial curve
CP_RaciPartial <- fRaciPartial$Ci(0)
```

ANALYSIS OF THE SLOW A-CI CURVE

Import slow file

```
httr::GET(url = paste0(testfile.url, "Slow.xlsx"), write_disk("Slow.xlsx", overwrite = TRUE))
```

```
Slow <- suppressMessages(read_excel("Slow.xlsx", skip = 14, sheet = "Measurements")) %>% select(A, Ci, CO2_r,
    gtc, gsw, E, Ca, Tleaf, Qamb_in) %>% slice(-1) %>%
    mutate_each(funs(as.numeric))

Slow <- select(Slow, Photo = A, PARi = Qamb_in, everything())
Slow$Rd <- pull(Dark)
```

Fit slow A-Ci with plantecophys

```
faci <- fitaci(Slow, useRd =TRUE)
par(mgp=c(2.5,1,0), family="serif", ps=14)
plot(faci, las=1,xlim=c(0,1200), ylim=c(0,30))
```

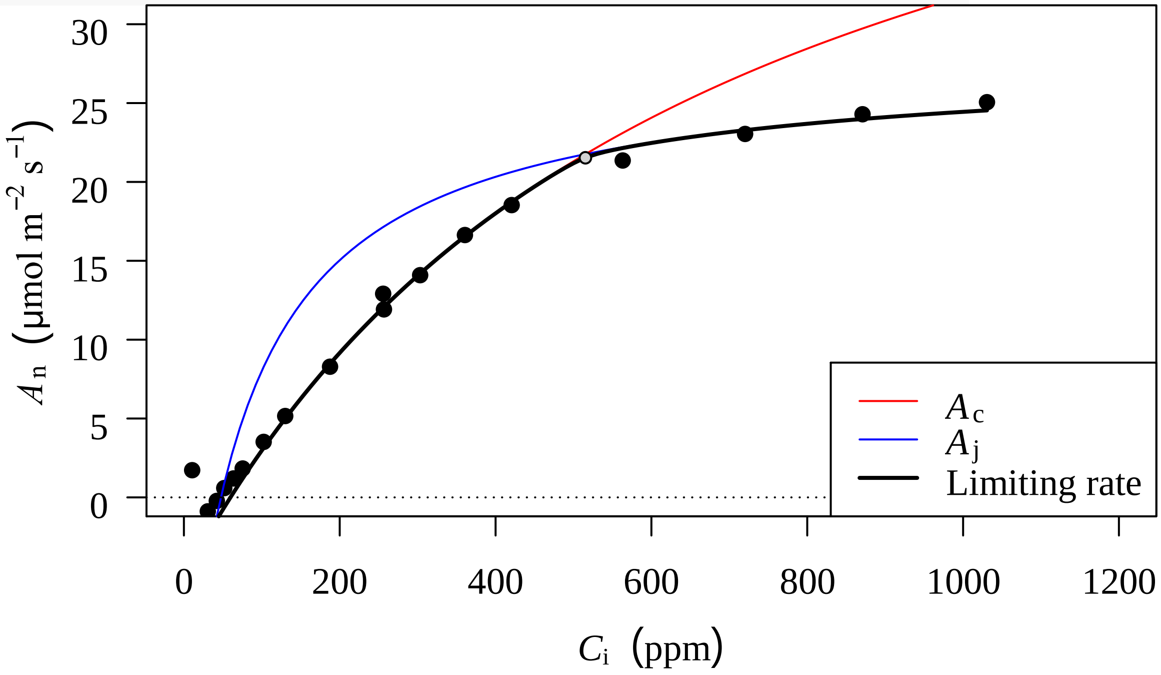

```
# Estimation of Co2 Compensation point
CP_Aci <- faci$ci(0)
```

ESTIMATES FOR THE THREE TEST FILE CURVES

	Vcmax	Vcmax_std	Jmax	Jmax_std	Rd	Rd_std	C_Point
Aci	63.69	2.69	138.35	5.63	1.522822	NA	59.77
Raci	64.73	0.081	147.61	0.15	1.522822	NA	59.21
Partial_Raci	65.12	0.07	144.67	0.17	1.522822	NA	59.09
